# Supplementary material for: Model-based analysis of the acute effects of transcutaneous magnetic spinal cord stimulation on micturition after spinal cord injury in humans
Source: PLoS Comput Biol. 2024 Jul 1;20(7):e1012237. doi: 10.1371/journal.pcbi.1012237 (PMC11244836; doi:10.1371/journal.pcbi.1012237)
Supplement: S1 Materials — Table A: Summary of model equations and terms used in Hodgkin-Huxley formulation of nodes. Table B Parameters and variables of Hodgkin-Huxley model. Table C: Parameters and variables of network of Hodgkin-Huxley model. Table D: Parameters and variable associated with TMS stimulation model. Table E: A summary of the axonal (cable) variables, parameters and differential equations used within the model. Table F: The nodal parameters and variables used within the model. (DOCX) [file pcbi.1012237.s001.docx]

Supplemental Material for PCOMPBIOL-D-23-01456 r1:

**Model-based Analysis of the Acute Effects of Transcutaneous Magnetic Spinal Cord Stimulation on Micturition after Spinal Cord Injury in Humans**

Mahshid Fardadi^1^, J.C. Leiter^2^, Daniel C. Lu^3^, and Tetsuya Iwasaki^1^

# Supplemental Material

# The Hodgkin Huxley Model of Neurons:

The model represents a network of interacting neural elements, and each element or node of the model was described as a single compartment Hodgkin-Huxley type model [1]. The effect of transcutaneous magnetic stimulation was modeled as an immediate activation of voltage-gated sodium channels in neurons in the lumbar spine that induced current flow into the neuronal soma. The governing equations for each neuronal element of the model are shown in Table A:

Table A Summary of model equations and terms used in Hodgkin-Huxley formulation of nodes.

| $C_{n}\frac{dV_{m}(j)}{dt}=I_{TMS}\left( j \right)-I_{K}\left( j \right)-I_{Na}\left( j \right)-I_{L}\left( j \right)-I_{SynE}\left( j \right)-I_{SynI}\left( j \right)$ |
| --- |
| in which, *j* is associated with the *j^th^* neuron in the network and: |
| $I_{Na}\left( j \right)=g_{Na}(v(j)-v_{Na})$ |
| $I_{K}\left( j \right)=g_{K}(v(j)-v_{K})$ |
| $I_{L}\left( j \right)=g_{L}(v(j)-v_{L})$ |
| $I_{TMS}\left( j \right)=g_{TMS}(v(j)-v_{Na})$ |
| The excitatory and inhibitory current for neuron *j*, can be calculated as follows: |
| $I_{SynE}\left( j \right)=\left\{ g_{SynE}.\sum_{i} \left[ S\left( w_{ij} \right).e^{-(t-t_{ki})/\tau_{SynE}} \right] \right\}.S(V(j)-E_{SynE})$ |
| $I_{SynI}\left( j \right)=\left\{ g_{SynI}.\sum_{i} \left[ S\left( -w_{ij} \right).e^{-(t-t_{ki})/\tau_{SynI}} \right] \right\}.S(V(j)-E_{SynI})$ |
| $V(j)$ is the membrane voltage; |
| $g_{SynE}$ and $g_{SynI}$ are the excitatory and inhibitory synaptic conductances. |
| $w_{ij}$ represents the weight of synaptic connection from neuron *i* to neuron *j* ($w_{ij}>0$for excitatory connections and $w_{ij}<0$ for inhibitory connections) |
| t_kj_ is the time each spike arrived at neuron i from neuron j, which increased the excitatory synaptic conductance by g_E_ [2] |
| $S\left( x \right)=\left\{ \begin{aligned} x, if x\geq0 \\ 0, if x<0 \end{aligned} \right.$ |
| The channel conductances in equation (1), $g_{Na}$ and $g_{K}$, varied according to the nonlinear dynamics represented by the latent variables m, h, and n: |
| $g_{Na}=\bar{g}_{Na}m^{3}h, m+\tau_{m}\left( v \right)\dot{m}=m_{\infty}(v)$ |
| $g_{K}=\bar{g}_{K}n^{4}, h+\tau_{h}\left( v \right)\dot{h}=h_{\infty}(v)$ |
| $g_{L}=\bar{g}_{L}, n+\tau_{n}\left( v \right)\dot{n}=n_{\infty}(v)$ |
| Where the variables *m, h,* and *n* are dimensionless numbers between 0 and 1 that represent the extent of sodium activation, sodium inactivation, and potassium activation, respectively. |
| The parameter values and the exact forms of $m_{\infty}(v)$, $\tau_{m}\left( v \right)$, etc. were as follows: |
| $\tau_{m}\left( v \right)=\frac{1}{\alpha_{m}\left( v \right)+\beta_{m}(v)} m_{\infty}\left( v \right)=\alpha_{m}(v)\tau_{m}(v)$ |
| $\tau_{h}\left( v \right)=\frac{1}{\alpha_{h}\left( v \right)+\beta_{h}(v)} h_{\infty}\left( v \right)=\alpha_{h}(v)\tau_{h}(v)$ |
| $\tau_{n}\left( v \right)=\frac{1}{\alpha_{n}\left( v \right)+\beta_{n}(v)} n_{\infty}\left( v \right)=\alpha_{n}(v)\tau_{n}(v)$ |
| Where the functions for each excitatory neuron were defined as follows [1]: |
| $\alpha_{m}\left( v \right)=\frac{0.182(v+35)}{1-e^{-{(v+35)}/9}} \beta_{m}\left( v \right)=\frac{-0.124(v-35)}{1-e^{{(v-35)}/9}}$ |
| $\alpha_{h}\left( v \right)=\frac{0.024(v+50)}{1-e^{-{(v+50)}/5}} \beta_{h}\left( v \right)=\frac{-0.0091(v-75)}{1-e^{{(v-75)}/5}}$ |
| $\alpha_{n}\left( v \right)=\frac{0.01(v+34)}{1-e^{-{(v+34)}/{10}}} \beta_{n}\left( v \right)=0.125e^{-{(v+44)}/{80}}$ |

The model variables and parameters are summarized in Table B.

Table B Parameters and variables of Hodgkin-Huxley model

| **Variable** | **Unit** | **Excitatory** | **Inhibitory** | **Description** |
| --- | --- | --- | --- | --- |
| i | ${\mu A}/{{cm}^{2}}$ |  |  | Current input per unit area |
| v | mV |  |  | Membrane potential |
| $\dot{v}$ | ${mV}/{ms}$ |  |  | Time derivative of membrane potential |
| C | ${\mu F}/{{cm}^{2}}$ | 1 | 1 | Electrical capacitance of the membrane |
| $\bar{g}_{Na}$ | ${k\Omega^{-1}}/{{cm}^{2}}$ | 30 | 35 | Sodium channel conductance |
| $\bar{g}_{K}$ | ${k\Omega^{-1}}/{{cm}^{2}}$ | 100 | 9 | Potassium channel conductance |
| $\bar{g}_{L}$ | ${k\Omega^{-1}}/{{cm}^{2}}$ | 0.1 | 0.1 | Leakage channel conductance |
| $v_{Na}$ | mV | 60 | 55 | Sodium channel reversal potential |
| $v_{K}$ | mV | -90 | -90 | Potassium channel reversal potential |
| $v_{L}$ | mV | -65 | -65 | Leakage channel reversal potential |
| $v_{rest}$ | mV | -65 | -64 | Resting (equilibrium) potential |
| $E_{Syn}$ | mV | -10 | -80 | Synaptic reversal potential |
| $g_{Syn}$ | ${mS}/{{cm}^{2}}$ | 0.08 | 0.12 | Synaptic channel conductance |

Table C Parameters and variables of network of Hodgkin-Huxley model

| **Neuron Location** | **Time constants(ms)** | **Connection weights,** $\boldsymbol{w}_{\boldsymbol{ij}}$ **from neuron i to neuron j** | |
| --- | --- | --- | --- |
| PMC | $\tau_{SynI}=5$ , $\tau_{SynE}=5$ | $w_{PAG-PMC}=0.25$ | N/A |
| PSC | $\tau_{SynI}=3$ , $\tau_{SynE}=3$ | $w_{5-PSC}=0.1$ | $w_{PMC-PSC}=-0.25$ |
| PAG | $\tau_{SynE}=1$ | N/A | $w_{5-PAG}=0.25$, $w_{HC-PAG}=0.35$ |
| HC | N/A | N/A | N/A |
| Neuron 1 | $\tau_{SynE}=5$ | N/A | $w_{PMC-1}=0.25$ |
| Neuron 2 | $\tau_{SynI}=5$ | N/A | $w_{PMC-2}=0.25$ |
| Neuron 3 | $\tau_{SynI}=3$ , $\tau_{SynE}=5$ | N/A | $w_{PSC-3}=0.09$ |
| Neuron 4 | $\tau_{SynI}=6$ , $\tau_{SynE}=1$ | $w_{2-4}=-0.4$ | $w_{8-5}=0.04$ |
| Neuron 5 | N/A | N/A | N/A |
| Neuron 6 | $\tau_{SynE}=5$ | N/A | $w_{3-6}=0.035$ |
| Neuron 7 | $\tau_{SynI}=35$ , $\tau_{SynE}=18$ | $w_{6-7}=-0.08$ | $w_{5-7}=0.13$ |
| Neuron 8 | $\tau_{SynI}=6$ , $\tau_{SynE}=10$ | N/A | $w_{7-8}=0.03$, $w_{1-8}=0.09$ |
| Neuron 9 | $\tau_{SynI}=1$ , $\tau_{SynE}=12$ | $w_{2-9}=-0.28$ | $w_{3-9}=0.05$, $w_{5-9}=0.13$ |

Table D Parameters and variable associated with TMS stimulation model.

| Variable | Unit | Excitatory | Inhibitory | Description |
| --- | --- | --- | --- | --- |
| k | N/A | 1/30 | 1/30 | Gain |
| $\tau$ | ms | 30 | 30 | Time constant for closing the sodium channel |

# Cable Model:

Within the model, axonal behavior of the pelvic nerve was modeled using axons represented by n nodes of excitable membranes connected in series by a cable. The cable element between nodes was called the internode. Each node was described by a Hodgkin-Huxley model, and the conductance of each internode was Gax. We used an (x,y) coordinate system where the origin was located at half of the node length to the left of the left end of the 1st node (the nodes were numbered 1,…,n from left to right).

The axial current, resulting from the external current injection Iext, acted as the input to the node, which was modeled by the Hodgkin-Huxley equations, as shown in Table E.

Table E A summary of the axonal (cable) variables, parameters and differential equations used within the model.

| $C_{n}\frac{dV_{m}(j)}{dt}=I_{axial}(j)-I_{ks}(j)-I_{Na}(j)-I_{L}(j)$ |
| --- |
| Each node, the channel currents were described as follows: |
| Persistant ${Na}^{+}$ current $I_{Nap}$: |
| $I_{Na}(j)=g_{Na}{m_{Na}\left( j \right)}^{3}h(j)(V_{m}(j)-E_{Na})$ |
| $\frac{dm_{Na}(j)}{dt}=\alpha_{mNa}(j)\left( 1-m_{Na}(j) \right)-\beta_{mNa}(j)m_{Na}(j)$ |
| $\alpha_{mNa}(j)={[-0.01(V_{m}(j)+60)]}/{[\exp\left( \frac{V_{m}\left( j \right)+60}{-10} \right)-1]}$ |
| $\beta_{mNa}(j)=4exp(\frac{V_{m}(j)+60}{-80})$ |
| $\frac{dh_{Na}(j)}{dt}=\alpha_{hNa}(j)\left( 1-h_{Na}(j) \right)-\beta_{hNa}(j)h_{Na}(j)$ |
| $\alpha_{hNa}(j)=0.07\exp\left( \frac{V_{m}\left( j \right)+60}{-20} \right)$ |
| $\beta_{hNa}(j)=exp(\frac{V_{m}(j)+30}{-10})$ |
| Slow $K^{+}$ current $I_{Ks}$: |
| $I_{Ks}(j)=g_{Ks}n^{4}(j)(V_{m}(j)-E_{K})$ |
| $\frac{dn(j)}{dt}=\alpha_{n}(j)\left( 1-n(j) \right)-\beta_{n}(j)n(j)$ |
| $\alpha_{s}(j)={[0.01(V_{m}(j)+50)]}/{[1-\exp\left( -\frac{V_{m}(j)+50}{10} \right)]}$ |
| $\beta_{s}\left( j \right)=0.125exp(\frac{V_{m}(j)+60}{-80})$ |
| Leakage current $I_{Lk}$: |
| $I_{Lk}=g_{Lk}(V_{m}(j)-E_{Lk})$ |

$X(j)$ and $Y(j)$ are the horizontal and vertical location of each node with respect to the coordinate system described earlier. For $X(j)$ we used the middle point of each node as the horizontal location of that node. Therefore,

$Y\left( j \right)=0$

$X\left( j \right)=Nodal_{L}*j+Internodal_{L}*\left( j-1 \right) j=1:node$

The model parameters and variables are defined as follows in Table F:

Table F The nodal parameters and variables used within the model.

| Nodal_L=1 $\mu m$ |
| --- |
| Internodal_L=1150 $\mu m$ |
| Number of nodes=21 ----- node=21 |
| Number of Internode=Number of nodes-1 |
| $\rho_{e}=500 \Omega cm$ |
| d: axonal diameter $d=6\mu m$ |
| L: internodal distance L$=1150\mu m$ |
| Nodal length=$1\mu m$ |
| $\rho_{ax}$: the axoplasmic resistivity $\rho_{ax}=70 \Omega cm$ |
| $E_{Na}=55.17 mV$ |
| $E_{K}=-72.14 mV$ |
| $E_{Lk}=-49.42 mV$ |
| $E_{rest}=-60 mV$ |
| $g_{Na}=1.2 mS/{cm}^{2}$ |
| $g_{Ks}=0.36 mS/{cm}^{2}$  $g_{Lk}=0.03 mS/{cm}^{2}$ |
| $C_{m}=0.01$F |
| $d=6 \mu m$ |

# Bibliography

1. Mahmud M, Vassanelli S. Differential Modulation of Excitatory and Inhibitory Neurons during Periodic Stimulation. Front Neurosci. 2016;10:62. Epub 2016/03/05. doi: 10.3389/fnins.2016.00062. PubMed PMID: 26941602; PubMed Central PMCID: PMCPMC4766297.

2. Danner SM, Wilshin SD, Shevtsova NA, Rybak IA. Central control of interlimb coordination and speed-dependent gait expression in quadrupeds. J Physiol. 2016;594(23):6947-67. Epub 2016/09/17. doi: 10.1113/JP272787. PubMed PMID: 27633893; PubMed Central PMCID: PMCPMC5134391.

**Table legends:**

**Table A:** Summary of model equations and terms used in Hodgkin-Huxley formulation of nodes.

**Table B** Parameters and variables of Hodgkin-Huxley model

**Table C**: Parameters and variables of network of Hodgkin-Huxley model

**Table D**: Parameters and variable associated with TMS stimulation model.

**Table E:** A summary of the axonal (cable) variables, parameters and differential equations used within the model.

**Table F:** The nodal parameters and variables used within the model.
