# Supplementary material for: Model-based analysis of the acute effects of transcutaneous magnetic spinal cord stimulation on micturition after spinal cord injury in humans
Source: PLoS Comput Biol. 2024 Jul 1;20(7):e1012237. doi: 10.1371/journal.pcbi.1012237 (PMC11244836; doi:10.1371/journal.pcbi.1012237)
Supplement: Attachment — Submitted filename: Review PCOMPBIOL-D-23-01456 Oct 2023.docx [file pcbi.1012237.s002.docx]

**Review of the paper:** *“Model-based Analysis of the Acute Effects of Transcutaneous Magnetic Spinal Cord Stimulation on Micturition after Spinal Cord Injury in Humans.”*

The authors describe in their manuscript a model to analyse the neural circuits controlling micturition after spinal cord injury and to evaluate through their simulations the effects of transcutaneous magnetic stimulation (TMS) as a therapeutic intervention. Their model is an updated (enhanced model as per their terminology) version of a pre-existing model and is built by adding pathways that improve the biophysical detailing. The enhanced model reproduces detrusor-sphincter dyssynergia, which is a typical pattern of bladder dysfunction after a spinal cord injury or SCI above the lumber level, by blocking or weakening inhibitory signals from the pontine storage center (PSC) and by strengthening the spinal voiding reflex. They model this effect by changing the synaptic weights among the interneurons regulating the spinal voiding reflex. In summary, the model simulates bladder dysfunction after spinal cord injury and attempts to shed light on possible mechanisms underlying the amelioration of detrusor-sphincter dyssynergia by TMS. Further, the model attempts to explore the complexity of interactions among lumbar interneurons in understanding the effects of spinal cord injury on bladder control.

**Comments**

1. The authors justify the need for the model to better understand through a system-level approach the mechanisms underlying micturition control or the deficits in it post an SCI. They explore possible pathways via which bladder control is improved via TMS. This study can also have interesting implications for the use of TMS in treating other conditions impacted by SCI.

2. While the work is interesting and of relevance, I feel the readability of the paper can be improved by developing better schematics of model interactions. For instance, the schematic shown in figure 1 can be augmented by showing a flow diagram of the model component interactions that map to the functional interaction shown in figure 1. In its current state, it is somewhat difficult to ascertain the complexity of the model (number of nodes, network density, number of excitatory/inhibitory inter neurons etc) owing to the lack of suitable flow diagrams and tabular data.

3. Another approach to improve readability can be to include tables. Some suggestions are as follows:

(i) a table of the model parameters in the (a) traditional model; and (b) enhanced model along with links to references (from experimental studies) from where model parameters have been obtained.

(ii) a second table summarising salient observations for the different states and conditions which are being simulated along with observations from experimental studies where such observations have been reported (if any).

(iii) The table can be further augmented to summarise the predictions from the model framework, once again tied to the experimental studies (if any) where such conjectures have been made. Improving readability will certainly enhance the understanding of the updated model and therefore improve the interpretation of the simulation results.

**Questions / Critique**

1. The authors model the effect of TMI pulses as an equivalent sodium conductance. Is there evidence of other kinds of channels (voltage-gated calcium channels for instance) present in the cellular processes at the nodes that participate in activating the neuron (excitatory or inhibitory)? Is there any other evidence of such activation apart from the comparison reported by the authors, i.e., neuronal soma in rat cortical brain slices? The authors need to shed light on the activation of these neurons (especially, from what is known from experimental studies).

2. The authors have reduced the model complexity by omitting the incorporation of post-ganglionic neuron dynamics into the model schema. Since an impact of neuronal firing especially post SCI can impact calcium dynamics at the synapses, could this omission mask some of the effects of SCI in evaluating detrusor responses?

3. Quantitative comparison of results (Figure 2): The authors report that the frequency of efferent hypogastric nerve action potentials increased from 10 to 45 Hz over 3s. They further report in Figure 2, that the parasympathetic activity underlying detrusor contraction was inhibited. Is there any quantitative comparison of these values with those obtained in experimental studies done by the authors (or a comparison with estimates that can be derived from experimental studies)? If yes, the authors should make such a comparison in their MS, as it will enhance the robustness / reliability of their findings.

4. Quantitative comparison of results (Figure 3): The results do provide a qualitative understanding of the process described after an SCI. Similar to Q3, are any quantitative estimates/comparisons possible for this set of results? Purely a qualitative account leaves one unconvinced about the robustness of the simulation outputs.

5. Can the authors explain quantitatively the translation of the two TMS frequency levels translate into equivalent activity in their model?

6. With reference to post-SCI neuroplasticity, it would be good if the authors comment on which components of the neural machinery are likely coding for this phenomenon. Furthermore, are these components explicitly coded into the model? If so, how?

**Overall observations**

(i) The paper reports an interesting model which studies interactions between different participants of the neural circuitry that are likely to impact detrusor activity post-SCI. It can therefore potentially serve as a framework to study conditions impacting detrusor activity post an SCI and to evaluate the efficacy of TMS by deriving insights into the mechanisms/reflex pathways that can be differentially activated by TMS based on its frequency.
(ii) **Comment for improvement:** Better schematics, quantitative comparisons or estimates and tabular data are missing and are needed to improve the readability of the paper. They will also help in ascertaining interactions between model components and better visualisation of the complexity/detailing of the model.
(iii) Experimental references are missing in certain instances, particularly in the supplementary section of the paper where model parameters have been reported. Wherever the authors have estimated model parameters or have used model tuning/fitting to obtain model parameters should be explicitly stated.
(iv) Note also [Important]: the Dropbox link to the code is not working. The authors should mention in the MS details regarding (i) the model implementation platform; (ii) numerical method used to solve the equations; (iii) initial condition values for the different state variables etc.

­Overall, the model has the potential to contribute to the field of study. However the model and its outputs need a good deal more explication, refinement, and quantitative grounding in order for such a paper to be acceptable for publication.
