# Supplementary material for: Model-based analysis of the acute effects of transcutaneous magnetic spinal cord stimulation on micturition after spinal cord injury in humans
Source: PLoS Comput Biol. 2024 Jul 1;20(7):e1012237. doi: 10.1371/journal.pcbi.1012237 (PMC11244836; doi:10.1371/journal.pcbi.1012237)
Supplement: Attachment — Submitted filename: Final version_ response to reviewers .docx [file pcbi.1012237.s003.docx]

Reviewer's Responses to Questions

**Comments to the Authors:**
Reviewer #1: This paper describes the use of a computational model to simulate the neural responses to TMS and the resulting bladder reflexes before and after SCI. While an important topic, the information in this paper is sometimes difficult to follow, in part due to inconsistent labeling and references between the text and figures.

**Response:** Thank you for your helpful comment. We revised the manuscript to use consistent labels and references between text and figures.

Introduction

Page 5: Clarify the phrase “Epidural stimulation and transcutaneous magnetic…” to be “Epidural electrical stimulation and transcutaneous magnetic…”

**Response:** Done.

In the second paragraph, the first sentence suggests that possible mechanisms for control will be discussed. Instead, existing computational models are discussed, without much justification regarding why these models are useful. I would like to see first a discussion of the key relevant reflexes, then an explanation of why the model presented here will add valuable insights to this field.

**Response:** The introduction has been restructured to describe more accurately the relationship of the current study to previous work. Because this comes up in later comments, we also tried to make it clear that previous investigators stipulated the simplest, most restricted set of elements controlling micturition that sufficed to replicate the behaviors being modelled. We have done the same. None of the previous models has been a comprehensive description of bladder control, and our ‘enhancement’ has been minimalist as well. We added the smallest possible number of new elements to existing models needed to replicate behaviors and responses that were not previously reproduced by earlier models. This design philosophy has now been made clear in the introduction, and where necessary, this design philosophy is emphasized in other parts of the paper.

Methods/Figure 1.: 1A. This figure suggests that the hypogastric nerve does not contain afferents and neglects the bladder neck connectivity of the hypogastric nerve. The “dot” ending of the branch off of the pelvic nerve is unlabeled and unclear; it appears to connect to the ureters in the figure. The green connection to the bladder is unlabeled (is this a limb of the pelvic nerve that has been separated out?) The nodes are colored but there is no key for those colors, and there is no key for the connection colors, which are difficult to see because the lines are very thin. Additionally, it should be noted that these color choices are essentially impossible to distinguish if one is colorblind. I think the best way to address the node issue here is to color them the same color, and number them all; this way they can be referred to consistently between figures 1A and B.

**Response:** Figure 1 was adapted from another publication, and some of the elements requested were not included in the original figure (or model). The original figure emphasizes the efferent control of bladder activity. We are trying to strike a balance between the minimum elements necessary to replicate a behavior within a mathematical model, and the diversity of inputs and outputs that exist in the natural state. In the same spirit, we are trying to provide complete information in the figure, but adding too many elements makes the figure too busy to interpret. Having said that, we revised the figure(s) along the lines suggested by the reviewer, and added test to the legend to make it clear that afferent activity exists in many of the nerves, but it is not shown in the figure. There are no ureters in the figure. The color code is red for urine storage, green for urine emptying and blue if the nerve is involved in both processes (e.g., the hypogastric and pudendal nerves). This is stated in the text. In addition, we added information to the text making it clear that the model we developed has more interneurons than models that went before, but it is still a minimalist representation of the physiology of micturition (which is relevant to comments below as well).

The description of this figure in the text is pretty confusing. The text describes activity at the lumbosacral spinal cord, which is not a region of the spinal cord labeled in the figure. In the text describing of Figure 1A, the authors state: “This bladder-derived afferent information provides a ‘tonic influence’ to activate the PSC, which in turn stimulates neurons in the Nucleus of Onuf that contract muscles of the pelvic floor and the external urethral sphincter. The muscles of the pelvic floor and the external urethral sphincter are also actively contracted during urine storage by efferent activity originating from spinal interneurons that respond to afferent information transmitted from the bladder and urethra through pelvic and pudendal nerves, respectively.” However, the pelvic floor is not labeled in the figure, and the innervation of the pelvic floor is unclear in the literature (in animals, it is innervated by the levator ani nerve; in humans it may be innervated by the levator ani or the pudendal) and the direct involvement of the pelvic floor in LUT reflexes is unclear. Further, activity of the pelvic floor is not directly modeled—it may be unnecessary to mention it. The urethra is not labeled in the figure and it is not clear in the text that activity in the urethra is carried by the pudendal nerve.

**Response:** Some of the confusion originates from our attempt to do justice to the complexity of the underlying physiology in the native state while making the point that we have made a minimalist representation of the physiology (and the figure is already pretty busy). We revised this section and, as part of responding to the request to restructure the introduction, made it clearer what is in the model and what is present in the native state but not represented in and not necessary for the model (because we were able to simulate more complex behaviors following spinal cord injury without those elements, even though they may contribute information (to some extent redundant) to the processes that are in the model). Hence, we have retained a brief mention of the structural and functional elements of micturition that are not in the model, but made it clear what is in the model, made it clear that our approach represents the minimum necessary elements to replicate the behaviors being modelled, and labelled the figure more completely – all within the spirit of the reviewers request.

Figure 1B, similarly to 1A, would benefit from additional legend elements and simplification of the color scheme. For example, on page 8, a reference is made to the “red circuits, excluding the projection to Node 7.” I was confused by this description – it looks like that’s the color for the pelvic nerve, and node 2 is also red but not touching any red neurons, and there are a couple of red interneurons, but they don’t all connect to each other.

**Response:** Revised to enhance clarity – we made what was dark red, more clearly a bright red. We tried to clarify connectivity and the role of each node clearer in the text.

Page 8 paragraph 2: I’d like a citation for the statement that the lack of PMC descending signals is what causes discoordination of detrusor and EUS.

**Response:** It is clear that the uncoordinated activation of the detrusor and EUS emerges after SCI, and the PMC coordinates these elements of the bladder when the spine is intact. So we reworded this sentence to ‘allows uncoordinated (activation) ….to emerge.’ We hope that addresses the intended purpose of the comment.

The framing of the last sentence suggests that overactive bladder is the cause of DSD; rephrase this.

**Response:** Revised as requested.

Page 8 paragraph 3 to page 9: By “added lumbar interneurons” – is there a physiological basis for the specific things you added or is it just something arbitrary added to make the model cooperate? Where are they in your figure in terms of node numbers (“shown in blue” is unclear given that there are multiple colors of blue)?

**Response:** First, the lumbar interneurons were added as a mechanism of getting the model ‘to cooperate’ based on replicating the behaviors described in the physiological studies of bladder function following cord transection. The additions made to the model are not arbitrary, De Groat et al speculated what the plausible neurophysiological basis of the reconstitution of the neonatal spinal voiding reflex would require [1], and we confirmed that the proposed mechanisms, when incorporated into a mathematical model would (largely) replicate the proposed role of the spinal voiding reflex in post-SCI bladder behavior. Threshold-dependent bladder emptying appears after SCI, and this has been attributed to restoration of an infantile reflex mechanism. In order to replicate this in a mathematical model, the elements that we added to the model were necessary, but also physiologically plausible based on previous animal work. To turn the question around, what is the alternative? Interneurons exist in the proper locations in the spine below the level of injury; they manifest the appropriate neuronal behaviors necessary to replicate the observed behaviors. There are no more obvious candidates to effect the necessary changes in control of micturition to reproduce the observed behaviors after SCI. We explained this more clearly through the manuscript. Any added lumbosacral interneurons were numbered, and references to these blue neurons have been made clearer in the text (there are only two nodes shown in blue, and they are in the PMS).

What is the justification for each added interneuron? Again, nothing in the figure is labeled “lumbar;” please be consistent with descriptions between the figure and text to keep it clear.

**Response:** See above. The justification for each interneuron has been explained more fully in the text. The labelling of the figure has been improved as suggested by the reviewer by labelling the lumbar and sacral sections separately in the schematic representations of the spine.

“The other node within the PMC” – please just describe by node numbers and include a figure ref to 1B here.

**Response:** Revised by making the reference clearer.

This is the first mention of social appropriateness, but I don’t see any results relevant to changes in your model at these higher centers; additionally the statement about how social appropriateness is controlled is not cited.

**Response:** This statement has been deleted at this point in the manuscript.

“Bladder filing - indicate” should be “bladder filling, indicating.”

**Response:** Revised as suggested.

The green arrow described here is a little confusing because there are multiple green connections in the figure.

**Response:** Revised by pointing out that it is the green arrow outside the spine. Throughout we have tried to increase the clarity of what the text is referring to in each figure.

Page 10: How did you decide on the influence of applied stimulation? Do you know how diffusely TMS spreads? It may activate more than just the interneurons described here, such as direct activation of the pelvic, pudendal, or hypogastric nerves. Specify the frequencies that this stimulation was applied at. Do you expect that TMS would have a frequency-dependent effect through the same mechanisms as nerve stimulation?

**Response:** We do not have exact answers to these questions. This is what we do know. There was a frequency-specific effect to TMS applied to the lumbar spine in humans: 30 Hz stimulation seemed to favor EUS activation and detrusor relaxation (enhancing urinary continence) and 1 Hz stimulation favored detrusor activation and EUS relaxation and coordinated bladder emptying [2]. For this reason, 1 and 30 Hz were the stimulation frequencies applied in the model. We do not believe that TMS spreads into the surrounding tissue. In order to achieve bladder-related effects in humans, we used the TMS at near maximal intensity (60-80% of maximal power - the maximum was set by the tolerance of each subject, and the tolerance was limited by contraction of the superficial muscles along the spine in the lumbar region). Lower levels of stimulation did not modify bladder function during the video-cystoscopy. In addition, the stimulation was applied at L1, and though not described in the paper, stimulation above or below that level was ineffective, which argues against stimulation of peripheral nerves as a mechanism of the TMS effects. We conclude from this that the TMS must penetrate the spine since we required near maximum power TMS was necessary to see an effect. Within the spine, we agree many neurons might be affected, but from the frequency-dependent effects, we infer that the relative excitation of inhibitory and excitatory neurons favored inhibitory neurons at the lower frequency of stimulation. This is a simplified, reduced model, and we only needed to add three interneurons to the network to simulate the bladder responses to TMS that we had previously observed. We added these clarifying points to the discussion along the lines indicated by the reviewer. Finally, the proposed electrophysiological neuronal responses to TMS are consistent with the available literature (which is small), as cited in the discussion.

Page 11: I am concerned about the description of the changes to the “traditional circuit” described in the second paragraph on page 11 (“The ‘traditional circuit’ has been simplified as shown in Figure 1A in that sphincter afferents in the pudendal nerve are not considered…the effect of pudendal afferent information is, to some extent, redundant with information from the bladder”). While the pelvic afferents are often described (by deGroat and others) as the primary drivers of LUT reflexes, stimulation on the pelvic and pudendal afferents can evoke different responses. The pelvic afferents, when stimulated, tend to produce a bladder contraction which may be accompanied by a sphincter contraction. The pudendal afferents, when stimulated, can produce a frequency-dependent response, as described by McGee (ref 12 in this paper). Describing them as redundant is therefore incomplete, and this description minimizes the potential impact of other neural populations.

**Response:** We revised as suggested. We did not model the pudendal afferent effects, and other than stating this plainly, the less said the better.

Page 13: In the first paragraph, change “seems reasonable” to something like “Acute experiments suggest that this mechanism can approximate…”

**Response:** Revised as suggested.

Results 3.1/Figure 2.: In the text, the circuit elements in 2A/B are described as “highlighted” but it’s difficult to discern what this includes in the figure, please clarify this.

**Response:** Revised the figure to make the cited elements clearer.

Figure 2C-D axis labels are hard to read; use consistent font style and size. It’s unclear where these voltages are recorded from – specifically label that it is membrane potential from a certain node for each y axis in C1-D3.

**Response:** Revised as suggested, and the legend has been updated to make it clear that neuronal activity is derived from changes in nodal membrane potential.

Results 3.2-3.3/Figure 3.: Axis label and figure lettering style has changed from Figure 2, please be consistent. Figure components are misaligned. No y-axis label.

**Response:** Revised as suggested; labels added and aligned as suggested.

Why do you think C1 shows a rapid firing at the onset of the simulation?

**Response:** The firing rate was determined by the attributes of the ionic currents and the synaptic currents. In this particular case, the firing rate was driven by afferent activity in the pelvic nerve, which varied across a broad range depending on the extent of bladder filling.

Results 3.4/Figure 4.: The description of the application of TMS to the circuit should likely be in the Methods section, rather than the Results. Labels A-D are in yet a different format compared to previous figures.

**Response:** Revised as suggested.

Results 3.5/Figure 5.: Is figure 5A-B presenting any new information? It seems to be replicating earlier figures and I don’t see the purple highlight described in the text. Figure 5C is not referred to in the text; the text does refer to a figure 5 E that doesn’t seem to exist so you may have gotten your numbering off by one. Please label axes; lack of labels and mislabeled figure numbers makes it difficult to offer feedback on the results in this section.

**Response:** Revised/corrected as suggested.

Figure 6 appears not to be referenced in the text and the font size is too small to easily read labels.

**Response:** We decided to drop Figure 6. It was meant to supplement the description of the Hodgkin-Huxley elements of the model. But axonal modeling is well established and clear in the code. So the figure is superfluous.

Discussion

Page 20-21: Two key findings are described: First, that there is an important infantile spinal voiding reflex, and second, that the model must contain excitatory and inhibitory interneurons with different sensitivity. The first seems to me to be less of a significant finding and more of a metric that was used to validate the model, as it is well-established that this reflex exists and is relevant to changes after SCI. The second would be more convincing with additional justification of the reasoning behind the components that were added to this model compared to a “traditional” model.

**Response:** First, we agree that these are the two key additions to the traditional model. Calling the implementation of the spinal voiding reflex a metric seems a little off target, but demonstrating that the minimal additions to the traditional model necessary to replicate the behavior is a validation of the putative neurophysiological mechanisms put forward earlier to explain the re-emergence of the reflex after SCI [1]. The discussion of this finding has been revised along the lines suggested by the reviewer. We agree that this reflex exists, but it has never been incorporated explicitly into a mathematical model, so far as we know. The mathematical formulation of the spinal voiding reflex, as we present it, is the simplest (and therefore most likely correct) configuration of the neural elements that permit the reflexes to emerge from the mathematical model. As the reviewer has suggested above, we have tried to make this logic clearer throughout the paper.

Given that the post-SCI emergent spinal voiding reflex is activated by C fibers rather than A-delta fibers, is it reasonable to build a model that represents both of those fiber populations as the same and suggests that all of the changes after SCI are due only to interneuron populations and descending signals?

**Response**: The reviewer is correct, and this is an excellent question. Within both the traditional and our enhanced model, there is no differentiation of A- or C-fiber afferents to replicate the observed behaviors. The models are based on frequency-dependent afferent information that is not differentiated as to receptor mechanisms or axonal conduction velocities. We and others have proposed the simplest mathematical circuit that reproduces the physiological responses after SCI. As such, these models represent a starting point for further modeling that should become increasingly accurate and representative of the neural structures underlying the control of micturition. We are discussing further elaborations of the model in which additional sensory and motor control elements would be added, and the sensory input could be modified to reflect A- and C-fiber inputs separately in the next iteration of a more comprehensive model. But this is beyond the scope of this first model. We add a comment to the limitations of the methods to reflect the reviewer’s comment.

Page 24: Remove the extra parenthesis after “stimulation of the detrusor.”

**Response:** Done.

It’s worth noting that citation 61, here used to describe the downfalls of “afferent or efferent arms of reflex control of the bladder,” primarily discusses tibial and pudendal nerve stimulation, neither of which is considered in this model.

**Response:** Duly noted. The sentence was deleted; the more general statement of the problem in the preceding sentence is probably sufficient for the discussion of this limitation.

Can you expand on the statement “The differential sensitivity of excitatory and inhibitory interneurons to TMS in the model may explain why low-frequency TMS treatment more effectively restored voluntary control of micturition than 30 Hz TMS?” This statement seems to be one of the key arguments in this paper but I’d like a walkthrough of exactly how the model explains this.

**Response:** The explanation of this point has been expanded slightly in the discussion. It is a key argument and a necessary part of the model to reconcile the model results with the response to TMS that we previously observed in patients with SCI. We cited the (small) relevant literature, but this topic has not been well studied. So we cannot go much beyond what we already said.

6.1 The site for BioRender should be “BioRender.com” not “BioRenders.com.”

**Response:** Corrected.

**Reviewer 2**

**Review of the paper:** “Model-based Analysis of the Acute Effects of Transcutaneous Magnetic Spinal Cord Stimulation on Micturition after Spinal Cord Injury in Humans.”

The authors describe in their manuscript a model to analyse the neural circuits controlling micturition after spinal cord injury and to evaluate through their simulations the effects of transcutaneous magnetic stimulation (TMS) as a therapeutic intervention. Their model is an updated (enhanced model as per their terminology) version of a pre-existing model and is built by adding pathways that improve the biophysical detailing. The enhanced model reproduces detrusor-sphincter dyssynergia, which is a typical pattern of bladder dysfunction after a spinal cord injury or SCI above the lumber level, by blocking or weakening inhibitory signals from the pontine storage center (PSC) and by strengthening the spinal voiding reflex. They model this effect by changing the synaptic weights among the interneurons regulating the spinal voiding reflex. In summary, the model simulates bladder dysfunction after spinal cord injury and attempts to shed light on possible mechanisms underlying the amelioration of detrusor-sphincter dyssynergia by TMS. Further, the model attempts to explore the complexity of interactions among lumbar interneurons in understanding the effects of spinal cord injury on bladder control.

Comments

1. The authors justify the need for the model to better understand through a system-level approach the mechanisms underlying micturition control or the deficits in it post an SCI. They explore possible pathways via which bladder control is improved via TMS. This study can also have interesting implications for the use of TMS in treating other conditions impacted by SCI.
2. While the work is interesting and of relevance, I feel the readability of the paper can be improved by developing better schematics of model interactions. For instance, the schematic shown in figure 1 can be augmented by showing a flow diagram of the model component interactions that map to the functional interaction shown in figure 1. In its current state, it is somewhat difficult to ascertain the complexity of the model (number of nodes, network density, number of excitatory/inhibitory inter neurons etc) owing to the lack of suitable flow diagrams and tabular data.

3. Another approach to improve readability can be to include tables. Some suggestions are as follows:

(i) a table of the model parameters in the (a) traditional model; and (b) enhanced model along with links to references (from experimental studies) from where model parameters have been obtained.

**Response:** The traditional model does not exist as a comparable mathematical formulation; it exists largely as a set of figures or simplified logic circuits. It is not, for that reason, directly comparable with a table of model parameters. In addition, there a no single cell recordings (current clamp or patch clamp) to identify unique ionic currents in the neurons that support micturition. Therefore, we used typical conductances in the Hodgkin-Huxley formulations used for each node. There is nothing bladder-specific about the neurons in the model, and others have faced the same issue when modeling aspects of bladder function [3]. This is actually an interesting point, and we appreciate that the reviewer brought it up. We explained this more fully in the text and provided a justification for the logic of doing this and the model parameters selected – that non-specific neuronal formulations at the nodes of the model can simulated the control of micturition indicates that the pattern of connectivity and the synaptic connections (inhibitory or excitatory) are the important elements of bladder control, which is consistent with the experimental literature cited in the paper.

(ii) a second table summarising salient observations for the different states and conditions which are being simulated along with observations from experimental studies where such observations have been reported (if any).

**Response:** We agree with the need for greater clarity among the multiple states that we modelled. Based on comments from Reviewer 1, we tried to fulfill the spirit of this request by adding more section headings that break the modeling process into its parts and adding clarifying text rather than a table.

(iii) The table can be further augmented to summarise the predictions from the model framework, once again tied to the experimental studies (if any) where such conjectures have been made. Improving readability will certainly enhance the understanding of the updated model and therefore improve the interpretation of the simulation results.

**Response:** We tried to map experimental observations onto model features and then map these features on to key model outputs by making the steps of developing the model clearer and labelling the sections using more informative language.

Questions / Critique

1. The authors model the effect of TMI pulses as an equivalent sodium conductance. Is there evidence of other kinds of channels (voltage-gated calcium channels for instance) present in the cellular processes at the nodes that participate in activating the neuron (excitatory or inhibitory)? Is there any other evidence of such activation apart from the comparison reported by the authors, i.e., neuronal soma in rat cortical brain slices? The authors need to shed light on the activation of these neurons (especially, from what is known from experimental studies).

**Response:** There is no evidence of activation of other channels that we are aware of, especially not from spinal neurons. One of the reasons to use mathematical models is to guide the next set of experiments, and as the reviewer is pointing out, experiments to develop more specific information about TMS effects on spinal neurons would be valuable.

2. The authors have reduced the model complexity by omitting the incorporation of post-ganglionic neuron dynamics into the model schema. Since an impact of neuronal firing especially post SCI can impact calcium dynamics at the synapses, could this omission mask some of the effects of SCI in evaluating detrusor responses?

**Response:** We do not believe so. The post-ganglionic dynamics do not add anything that would fundamentally change the response (they do not, for example, reverse the sign of activity at any point in the model (excitation remains excitation through the simplifications, for example). The details (actual firing rates, for example) might be slightly different, but not the general description of the response that emerges from the model. Nevertheless, we added this issue to the limitations of the methods within a general statement that the model is a minimal representation of the control of micturition, and further elaboration of the model including more realistic mathematical representation of the neurons (as suggested by Reviewer 1 as well) would increase the accuracy and detail of the predicted results. The issue of calcium dynamics is mentioned as well as a target of future modeling, especially in relation to model plasticity.

3. Quantitative comparison of results (Figure 2): The authors report that the frequency of efferent hypogastric nerve action potentials increased from 10 to 45 Hz over 3s. They further report in Figure 2, that the parasympathetic activity underlying detrusor contraction was inhibited. Is there any quantitative comparison of these values with those obtained in experimental studies done by the authors (or a comparison with estimates that can be derived from experimental studies)? If yes, the authors should make such a comparison in their MS, as it will enhance the robustness / reliability of their findings.

**Response:** We are not aware of any quantitative studies at the neuronal level – here again, the results of the model point to interesting experiments that could be done to increase our understanding of the neuronal processes that lead to the macroscopic reflex behaviors that have been observed. A comment to this effect has been added to the MS.

4. Quantitative comparison of results (Figure 3): The results do provide a qualitative understanding of the process described after an SCI. Similar to Q3, are any quantitative estimates/comparisons possible for this set of results? Purely a qualitative account leaves one unconvinced about the robustness of the simulation outputs.

**Response:** Similar response - not that we are aware of.

5. Can the authors explain quantitatively the translation of the two TMS frequency levels translate into equivalent activity in their model?

**Response:** The two frequencies of TMS do not translate into equivalent activities in the model, nor did they in prior human studies. The lower frequency of stimulation is much more effective eliciting activity in the inhibitory neurons in the lumbar region of the model, and this was associated with greater coordination between the detrusor and the EUS. Higher frequency stimulation seemed to activate the EUS and favored continence. This differential effect is based on reports from the literature, and incorporating these frequency-of-stimulation-dependent neuronal responses into the model produced differential responses in the firing patterns of the model that replicated the divergent behavioral responses to the two frequencies of TMS that we observed in humans. We tried to make this clearer in the MS.

3. With reference to post-SCI neuroplasticity, it would be good if the authors comment on which components of the neural machinery are likely coding for this phenomenon. Furthermore, are these components explicitly coded into the model? If so, how?

**Response:** The neuroplasticity that allows the infantile reflex control of bladder function to emerge is coded explicitly in the model in multiple ways that first establish the reflex, and then establish its inhibition to replicate bladder behavior in normal, intact adults. Nodes 6 and 7 are not explicitly in the traditional model of micturition control, and they were added to the model to allow the model to replicate the observed changes in bladder control seen after SCI. Moreover, the PMC and lumber interneurons were modified or added so that the spinal voiding reflex could be inhibited when the spine is intact. The behavior after simulated SCI reflects the predicted behavior of elements that were incorporated to simulate the normal response when the circuitry for the spinal voiding reflex exists, but is inhibited. We added further clarification of this point to the MS.

With respect to the longer term plasticity that we observed in humans (the restoration of partial volitional control of micturition after weeks of TMS treatment), we speculated, but briefly. This is a tougher nut to crack, and we are still thinking about what would be necessary.

Overall observations

(i) The paper reports an interesting model which studies interactions between different participants of the neural circuitry that are likely to impact detrusor activity post-SCI. It can therefore potentially serve as a framework to study conditions impacting detrusor activity post an SCI and to evaluate the efficacy of TMS by deriving insights into the mechanisms/reflex pathways that can be differentially activated by TMS based on its frequency.
(ii) **Comment for improvement:** Better schematics, quantitative comparisons or estimates and tabular data are missing and are needed to improve the readability of the paper. They will also help in ascertaining interactions between model components and better visualisation of the complexity/detailing of the model.

**Response:** We modified the figures as suggested by both reviewers, and we added clarifying headings or text to better visualize the complexity of the model – as suggested.

(iii) Experimental references are missing in certain instances, particularly in the supplementary section of the paper where model parameters have been reported. Wherever the authors have estimated model parameters or have used model tuning/fitting to obtain model parameters should be explicitly stated.

**Response:** This information is often simply not available. However, we provided additional justifications for the selection of the model parameters, as suggested.

(iv) Note also [Important]: the Dropbox link to the code is not working. The authors should mention in the MS details regarding (i) the model implementation platform; (ii) numerical method used to solve the equations; (iii) initial condition values for the different state variables etc.

**Response:** We apologize for this. We will comply with the journal policy, and all the code will be available to readers.

Overall, the model has the potential to contribute to the field of study. However the model and its outputs need a good deal more explication, refinement, and quantitative grounding in order for such a paper to be acceptable for publication.

**Response:** We complied with both reviewers’ requests for improvement, and we hope this will suffice to improve the publication.

1.	de Groat WC, Kawatani M, Hisamitsu T, Cheng CL, Ma CP, Thor K, et al. Mechanisms underlying the recovery of urinary bladder function following spinal cord injury. Journal of the autonomic nervous system. 1990;30 Suppl:S71-7. Epub 1990/07/01. doi: 10.1016/0165-1838(90)90105-r. PubMed PMID: 2212495.

2.	Niu T, Bennett C, Keller T, Leiter JC, Lu DC. A Proof-of-Concept Study of Transcutaneous Magnetic Spinal Cord Stimulation for Neurogenic Bladder. Sci Rep. 2018;8:12549.

3.	McGee MJ, Grill WM. Modeling the spinal pudendo-vesical reflex for bladder control by pudendal afferent stimulation. Journal of computational neuroscience. 2016;40(3):283-96. Epub 2016/03/13. doi: 10.1007/s10827-016-0597-5. PubMed PMID: 26968615; PubMed Central PMCID: PMCPMC4860361.
