# Supplementary material for: Model-based analysis of the acute effects of transcutaneous magnetic spinal cord stimulation on micturition after spinal cord injury in humans
Source: PLoS Comput Biol. 2024 Jul 1;20(7):e1012237. doi: 10.1371/journal.pcbi.1012237 (PMC11244836; doi:10.1371/journal.pcbi.1012237)
Supplement: Attachment — Submitted filename: Response to reviewers R2 Final.docx [file pcbi.1012237.s004.docx]

When you are ready to resubmit, please upload the following:

[1] A letter containing a detailed list of your responses to the review comments and a description of the changes you have made in the manuscript. Please note while forming your response, if your article is accepted, you may have the opportunity to make the peer review history publicly available. The record will include editor decision letters (with reviews) and your responses to reviewer comments. If eligible, we will contact you to opt in or out.

**Response:** Responses to Reviewers enclosed.

[2] Two versions of the revised manuscript: one with either highlights or tracked changes denoting where the text has been changed; the other a clean version (uploaded as the manuscript file).

**Response:** A redlined and CLEAN versions of the revised (R2) manuscript have been submitted.

**Comments to the Authors:**

Reviewer #1: This paper covers an interesting model of TMS to activate neural circuits that target the bladder. In this revision, it has been substantially improved by edits to the text and figures. However, some changes, particularly to figures, are necessary to communicate the goals effectively. In particular, it is absolutely essential that the first figure/schematic figure that is repeated throughout the rest of the work is clear to readers in order to communicate the circuits and manipulations.

Introduction: Page 5: After the phrase “Muscle dynamics have been described by Hill equations,” clarify what those are and provide a citation.

**Response:** The explanation has been expanded, and citations have been provided.

Methods: Page 7: There is a missing reference error beginning here and for all the Figure numbers.

**Response:** The reference error has been corrected (we do not see this; it seems to be introduced during creation of the submission in the submission portal. I hope that it is fixed, but this may be up to the journal).

Figure 1.: 1A. This figure suggests that the hypogastric nerve does not contain afferents and neglects the bladder neck connectivity of the hypogastric nerve. The “dot” ending of the branch off of the pelvic nerve is unlabeled and unclear; it appears to connect to the ureters in the figure. The green connection to the bladder is unlabeled (is this a limb of the pelvic nerve that has been separated out?) The nodes are colored but there is no key for those colors, and there is no key for the connection colors, which are difficult to see because the lines are very thin. Additionally, it should be noted that these color choices are essentially impossible to distinguish if one is colorblind. I think the best way to address the node issue here is to color them the same color, and number them all; this way they can be referred to consistently between figures 1A and B.

Reviewer response to response: I understand what you are trying to convey in this figure (1A and 1B), but it does not stand alone yet.

**Response:** Figure 1 has been revised as suggested by the reviewer. The endings of the lines have been separated so that each connection is clear. The afferent pelvic nerve node has been properly marked. The ureters have been labeled. The text of the figure caption has been expanded and clarified. N.B.: Node 4 was on the wrong node; it has been moved back to the correct node where interactions with descending information were encoded in the model. The colors were meant to provide contrast; they have no mechanistic significance, and this is explained in the text. At this point, we have done what we can. We wanted to take advantage of the figure in Fowler et al., which is widely cited as a general reference describing the commonly accepted principles of bladder control, but some of the legacy of this figure leaks into our model in that we did not need to simulate each node in the Fowler scheme, and it is a nodal schematic – not a fully accurate representation of the population-based neural network. We have explained this in the text of the paper and in the captions to the figures and hope this will suffice.

After looking into the Fowler paper you used to generate this figure, I found that the branch from the pelvic nerve with the dot/circle marker in 1A was in fact just the T junction and cell body of the pelvic nerve afferents. However, in this figure, unlike the Fowler figure, it appears to be a separate connection. There are (unlabeled) ureters in the figure (the faded out tubes on the left and right of the bladder image); there is no need to remove them from the figure but the branch from the pelvic nerve that has the dot on the end appears to connect to it as-is. This is misleading; please either include the cell bodies of all cells or remove this one. It is unclear if the various nodes/circles drawn here represent cell bodies (as appears to be the case for ie the hypogastric neurons), brain regions (as appears to be the case for “Higher Centers”), or simply a local area of interest (as appears to be the case for the TMS zone in 1B, since presumably TMS did not spontaneously generate new cell bodies). Additionally, please add a figure legend that includes the color information of each axon. You also appear to be suggesting with this figure that the pelvic afferents (red) contribute to urine storage but not voiding. Work by deGroat and others suggests that this is untrue. Because the shapes of the ends of the neurons are critical for understanding the figure, please make sure they are not overlapping with each other or with the circles you have drawn.

**Response:** Redrawn as suggested by the reviewer – with limitations. Once we start with the Fowler schematic, we are stuck with their representation. They included only the afferent pudendal input, and we explained how we did the same and its significance. The pelvic afferent and efferent functions are embodied in separate representations in the Fowler scheme (and in our model); this is explained in the text of the MS and in the caption.

In the caption, I would recommend beginning with a sentence such as “A. The circuits for micturition and continence are both shown in this figure.” to clarify the overlapping nature. You also contradict yourself in this caption: you say that the only afferents that are relevant are pelvic, but you have drawn the pudendal neuron with the ending connection labeled as afferent in the key. Can you please additionally explain why the nodes/cell bodies/circles in the figure are colored differently and what those colors mean? I don’t see that information for all of the nodes in the figure itself or in the caption.

**Response:** The figure legend and figure have been revised as suggested by the reviewer. The nodal structure originates with Fowler et al, but it is also an accurate representation of how the computer model was built, as explained in the text. As noted above, the colors were added for contrast; they have no mechanistic significance.

Figure 1B – many of the items from the notes on 1A above would also improve 1B. In particular, overlapping elements make it difficult to follow some of the paths. I would add that I don’t understand how the pelvic nerve branches as shown here, or why there are suddenly two PMC nodes. You show the hypogastric activity emerging from the lumbar spine here and the thoracolumbar spine in 1A, please pick one and give a reference for the origin of the hypogastric efferents in the cord.

**Response:** The figure has been amended as the reviewer suggests, and we explained in the text of the MS and in the caption why the PMC was broken into two populations of neurons (to show targeting of distinct inhibitory and excitatory caudal neuronal populations).

Regarding the equations in this section, I would agree with the other reviewer that a table format could make it clearer what each term is and what the purpose of each equation is.

**Response:** We believe this refers to the supplementary information (there are only three equations in the body of the paper, which do not lend themselves to presentation in a single table as they deal with disparate parts of the model). We placed the equations in the supplementary information in a table format. We are not sure it is an improvement. The editor can decide whether to use the earlier version or the current (r2) version of the supplementary information.

Figure 2 (and remaining figures) – please make the letters indicating figure parts all black and remove the parentheses. Please include a key in the figure itself that shows the meaning of the different colored lines (I know it is in the caption, but it will be much easier to read with a key). I assume that the black, red, and blue lines have different y axes? If so, please indicate in some way what their starting potentials are or where zero is for each of the traces.

**Response:** Figure 2 was revised as suggested. The potentials are accurate as drawn: the inhibitory and excitatory nodes did not have the same resting potential (as explained in the text, they had different potassium conductances).

Figure 3 – I appreciate the highlighted thicker lines, but it is confusing that it is a different color than the original lines. Can you make the lines thicker in their original colors instead, or if you need these to be highlighted in a certain color, can you remove the thinner line or make it black/a neutral color? What is it that happens around 1800 ms that changes the behavior of so many of these nodes? Given that this is the figure referred to for the “Healthy” section, I would prefer that it doesn’t have the dotted line on the figure indicating an SCI.

**Response:** The dotted line has been removed from the healthy part of the figure, and the thick lines have been redrawn with a single color. The explanation of the stepwise bladder filling (the source of the change in activity at 1800 ms) has been expanded, and additional symbols have been added to the figure related to this point.

Discussion: It may be helpful, given the opening framing of the discussion, to provide a table with the differences between the traditional model and the novel model, and the reasoning for each addition. This would be easy to refer back to rather than needing to go through text line-by-line.

**Response:** A table summarizing the differences has been added to the paper.

I am glad you added a note on the limitation of different fiber types on page 22, but if someone isn’t aware of the different roles of different fiber types in control of the bladder they may not have enough information here. I’d like to see a comment (just one sentence) that specifically addresses the differing roles of A-delta and C fibers in control of the bladder before/after SCI to give context.

**Response:** Revised as requested.

Could you explain further and give some physiological data for the statement “prolonged bursting in the model that is likely achieved in actual neuronal tissue by networks of neurons”?

**Response:** Further information and clarification have been added indicating that the simplified representation of the circuit makes each neuron do the job of what is in reality is generated by populations of interacting neurons.

Reviewer #2: The authors have responded adequately to the queries, made relevant changes to their manuscript, and provided requisite clarifications.

The responses are acceptable. Apropos the larger point made regarding the sparsity of model parameters being extracted from experimental literature/work specific to the bladder, it is important to note that while bladder-specific data might be sparse in relation to the components of the circuitry modelled by the authors, it is nevertheless useful and instructive to construct frameworks such as the one introduced here, to derive insights into bladder control and function. Model parameter values are within physiological bounds and are typical for individual components of the cellular machinery. The predictions and analyses presented are therefore relevant.

**Response:** We incorporated the reviewer’s comment almost verbatim into the discussion as it captures our reasons for developing the model almost exactly.

Figure Files:

While revising your submission, please upload your figure files to the Preflight Analysis and Conversion Engine (PACE) digital diagnostic tool, [https://pacev2.apexcovantage.com](https://nam12.safelinks.protection.outlook.com/?url=https%3A%2F%2Fpacev2.apexcovantage.com%2F&data=05%7C02%7CJames.C.Leiter%40dartmouth.edu%7C34e1e30d0ec943e91fd908dc42c222b7%7C995b093648d640e5a31ebf689ec9446f%7C0%7C0%7C638458651394603272%7CUnknown%7CTWFpbGZsb3d8eyJWIjoiMC4wLjAwMDAiLCJQIjoiV2luMzIiLCJBTiI6Ik1haWwiLCJXVCI6Mn0%3D%7C0%7C%7C%7C&sdata=sS6Hlj9poeQMDcwPy1vjvi7ckRqRl4wpClPfL4lxJjE%3D&reserved=0). PACE helps ensure that figures meet PLOS requirements. To use PACE, you must first register as a user. Then, login and navigate to the UPLOAD tab, where you will find detailed instructions on how to use the tool. If you encounter any issues or have any questions when using PACE, please email us at [figures@plos.org](mailto:figures@plos.org).

Data Requirements:

Please note that, as a condition of publication, PLOS' data policy requires that you make available all data used to draw the conclusions outlined in your manuscript. Data must be deposited in an appropriate repository, included within the body of the manuscript, or uploaded as supporting information. This includes all numerical values that were used to generate graphs, histograms etc.. For an example in PLOS Biology see here: [http://www.plosbiology.org/article/info%3Adoi%2F10.1371%2Fjournal.pbio.1001908#s5](https://nam12.safelinks.protection.outlook.com/?url=http%3A%2F%2Fwww.plosbiology.org%2Farticle%2Finfo%253Adoi%252F10.1371%252Fjournal.pbio.1001908%23s5&data=05%7C02%7CJames.C.Leiter%40dartmouth.edu%7C34e1e30d0ec943e91fd908dc42c222b7%7C995b093648d640e5a31ebf689ec9446f%7C0%7C0%7C638458651394616852%7CUnknown%7CTWFpbGZsb3d8eyJWIjoiMC4wLjAwMDAiLCJQIjoiV2luMzIiLCJBTiI6Ik1haWwiLCJXVCI6Mn0%3D%7C0%7C%7C%7C&sdata=8p00hW3u9qPn7NDfmz71VAO4sENdr803VQcb8K%2FnEx8%3D&reserved=0).

Reproducibility:

To enhance the reproducibility of your results, we recommend that you deposit your laboratory protocols in protocols.io, where a protocol can be assigned its own identifier (DOI) such that it can be cited independently in the future. Additionally, PLOS ONE offers an option to publish peer-reviewed clinical study protocols. Read more information on sharing protocols at [https://plos.org/protocols?utm_medium=editorial-email&utm_source=authorletters&utm_campaign=protocols](https://nam12.safelinks.protection.outlook.com/?url=https%3A%2F%2Fplos.org%2Fprotocols%3Futm_medium%3Deditorial-email%26utm_source%3Dauthorletters%26utm_campaign%3Dprotocols&data=05%7C02%7CJames.C.Leiter%40dartmouth.edu%7C34e1e30d0ec943e91fd908dc42c222b7%7C995b093648d640e5a31ebf689ec9446f%7C0%7C0%7C638458651394629763%7CUnknown%7CTWFpbGZsb3d8eyJWIjoiMC4wLjAwMDAiLCJQIjoiV2luMzIiLCJBTiI6Ik1haWwiLCJXVCI6Mn0%3D%7C0%7C%7C%7C&sdata=veE%2BC9vFW9kffKPf7CdvmTzcKAnRfbCxnXUvK5%2BqxtQ%3D&reserved=0)

*In compliance with data protection regulations, you may request that we remove your personal registration details at any time.* [*(Remove my information/details)*](https://nam12.safelinks.protection.outlook.com/?url=https%3A%2F%2Fwww.editorialmanager.com%2Fpcompbiol%2Flogin.asp%3Fa%3Dr&data=05%7C02%7CJames.C.Leiter%40dartmouth.edu%7C34e1e30d0ec943e91fd908dc42c222b7%7C995b093648d640e5a31ebf689ec9446f%7C0%7C0%7C638458651394642521%7CUnknown%7CTWFpbGZsb3d8eyJWIjoiMC4wLjAwMDAiLCJQIjoiV2luMzIiLCJBTiI6Ik1haWwiLCJXVCI6Mn0%3D%7C0%7C%7C%7C&sdata=8aPsAqhl7I1LdWZgFsI25bezHCtdFg3FGrSdX7caBOc%3D&reserved=0)*. Please contact the publication office if you have any questions.*
